# Chronic obstructive pulmonary disease and risk of lung cancer: a meta-analysis of prospective cohort studies

**DOI:** 10.18632/oncotarget.20351

**Published:** 2017-08-18

**Authors:** Xinyue Zhang, Ning Jiang, Lijuan Wang, Huaman Liu, Rong He

**Affiliations:** ^1^ Department of Lung Disease, The First Clinic Medical College, Shandong Traditional Chinese Medicine University, Jinan, Shandong Province, China; ^2^ Department of Traditional Chinese Medicine, Maternal and Child Health Care of Shandong Province, Key Laboratory of Birth Regulation and Control Technology of National Health Family Planning Commission of China, Jinan, Shandong Province, China; ^3^ Department of Lung Disease, The Affiliated Hospital of Shandong Traditional Chinese Medicine University, Jinan, Shandong Province, China; ^4^ Department of Internal Medicine, The Affiliated Hospital of Shandong Traditional Chinese Medicine University, Jinan, Shandong Province, China

**Keywords:** chronic obstructive pulmonary disease, lung cancer, meta-analysis, relative risk

## Abstract

**Background:**

Studies exploring the association between chronic obstructive pulmonary disease (COPD) and lung cancer have yielded mixed results. We conducted a meta-analysis of the published prospective cohort studies to have a clear understanding about this association.

**Methods:**

We searched the MEDLINE and EMBASE databases from inception to December 31, 2016. Bibliographies were also reviewed for additional information. Random-effects model was used to calculate summary relative risk (SRR) and corresponding 95% confidence interval (CI).

**Results:**

Eighteen prospective cohort studies were part of this meta-analysis, involving 12,442 lung cancer cases with a median duration of follow- up of 5 years (range: 1.5–20 years). A history of COPD, emphysema or chronic bronchitis conferred SRRs of 2.06 (95% CIs: 1.50-2.85; n=14 studies), 2.33 (95% CIs: 1.56–3.49; n=4 studies) and 1.17 (95%CIs: 0.79–1.73; n=3 studies), respectively. Stratification by COPD severity yielded SRR of 1.46 (95% CIs: 1.20–1.76) for mild, 2.05 (95% CIs: 1.67-2.52) for moderate and 2.44(95% CIs: 1.73-3.45) for severe COPD, respectively. There were similar risk estimations for never and ever smokers. The SRR was statistically higher for squamous cell cancer than that for adenocarcinoma and for small cell cancer of the lung (P<0.05).

**Conclusion:**

This meta-analysis indicated a significantly increased risk of lung cancer for COPD, emphysema, but not for chronic bronchitis. For the prevention of lung cancer, it is of importance for early detection of COPD in lung cancer surveillance.

## INTRODUCTION

Lung cancer and chronic obstructive pulmonary disease (COPD) are two major public health problems. According to the Global Burden of Disease (GBD) 2013, these two diseases are among the six non-communicable leading causes of years of life lost globally [1]. World Health Organization estimated that COPD affects about 65 million people in the world, of which 5% died in 2005 and 90% of those deaths occurred in low and middle income countries [2]. In almost every country lung cancer is within the top leading causes of death [3]. It is estimated that by 2030 it will still be one of the main causes of death [4].

COPD is characterized by progressive and incompletely reversible airflow obstruction in the lungs. Epidemiological studies have observed an association between these two diseases and mechanisms are most likely related to systemic inflammation, oxidative stress, lung repair mechanisms [5]. Though the results were fairly heterogeneous, two meta-analyses till now have evaluated COPD as a risk factor for lung cancer [6, 7]. The most recent report included literatures up to August, 2010, which included 31 case-control and 8 cohort studies [8–15], and found the overall relative risks (RR) (95% confidence interval [CI]) for a previous history of COPD, chronic bronchitis or emphysema of 2.22 (1.66-2.97), 1.52 (1.25-1.84) and 2.04 (1.72-2.41), respectively [6]. Since that report was published, many prospective cohort studies on this issue have emerged [15–24]. Case-control studies [25–32] reported exposure information obtained after lung cancer diagnosis; these data thus be subject to recall bias and inaccurate assessments of COPD. Moreover, selection bias is a concern in case-control studies. Therefore, to obtain a more accurate and precise estimated effect of COPD on risk of lung cancer, we conducted a comprehensive meta-analysis of prospective cohort studies using our own methods and criteria in the selection of studies, in the presentation of data, and in our conclusions and interpretation of the evidence. This meta-analysis followed the guideline on meta-analysis of observational studies in epidemiology (MOOSE) [33].

## RESULTS

### Search results

**Figure 1:**
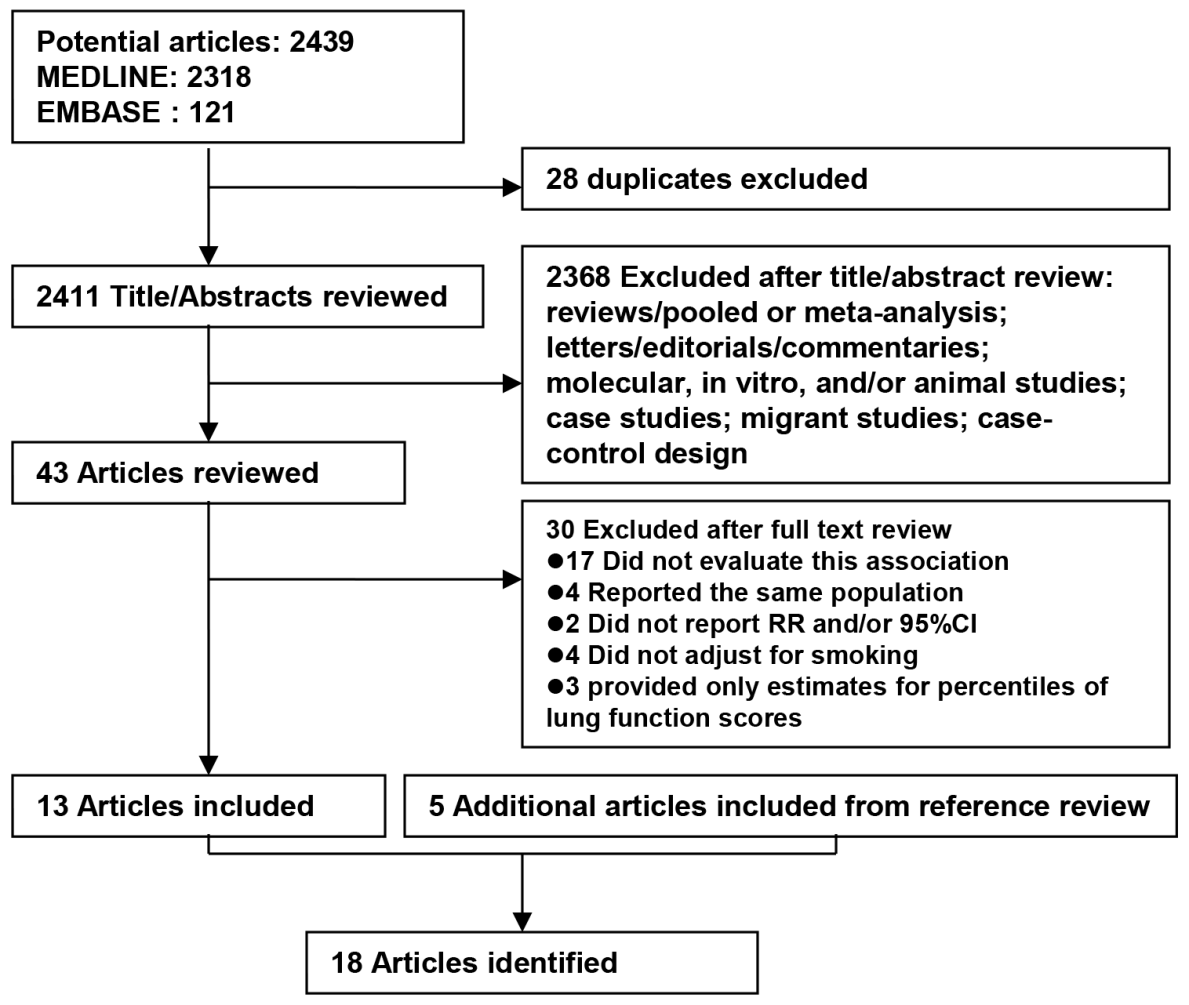
Flow diagram of systematic literature search on the association between chronic obstructive pulmonary disease and risk of lung cancer

Based on the study selection criteria, we identified a total of 2,439 potentially relevant articles (2,318 articles from MEDLINE database and 121 articles from EMBASE database). Among these 2439 articles, 72 were considered potentially relevant and their full texts were retrieved for further evaluation. Fifty-nine were excluded for various reasons: 17 did not evaluate this association, 4 reported the same population [10, 34–36], 2 did not report RR and/or 95%CI [37, 38], 4 did not adjust for smoking [39–42], and 3 provided only estimates for percentiles of lung function scores [8, 43, 44]. In addition, 5 more articles were identified by studying the cross-reference list. Therefore, a total of 18 cohort studies were used for this meta-analysis (Figure 1).

### Study characteristics

Table 1 depicted the characteristics of these studies. In a total of 931,627 of participants, 12,442 lung cancer cases were observed after a median duration of follow- up of 5 years (range: 1.5–20 years). The continents or countries in which the studies were conducted were as follows: China (n=3), the United States (n=9), and Europe (n=6). For the diagnosis of COPD, seven studies [12–14, 16, 18, 24, 45] used forced expiratory volume (FEV) or radiographic evidence of emphysema, eight studies [9, 11, 19, 20, 22, 23, 46, 47] based on self-reported condition from questionnaire, and three studies [15, 17, 21] used physician report, medical report or disease registry.

**Table 1:** Characteristics of prospective cohort studies of chronic obstructive pulmonary disease and lung cancer risk

Author/ year, Country	Cohort size and type Baseline age	COPD assessments	Duration or range of Follow-up, years	Type of outcome Cases (n)	RR (95% CI)	Adjustments
Oelsner et al./2016/USA [24]	MESA, N=6784 Age: 62 years; M(47.1%)	CT	12	Death, N=538	RR: 1.84 (1.09–3.12)	Age, sex, race/ethnicity, BMI, site, smoking, coronary artery calcium score and education
Marcus et al./2015/England [23],	LLPi, N=8760 Age: 45-79;M (47.8%)	Self-report HES database	8.7	Incidence N=237	HR: 2.43 (1.79–3.30)	Age, sex, smoking duration, prior diagnosis of pneumonia, asthma, prior diagnosis of malignant tumor, and family history of lung cancer
Aldrich et al./2015/USA [22]	SCCS, N= 26,927 Age: 45-79; M (33.1%)	CMS	2002-2009	Death, N=318	HR: 2.12 (1.59–2.82)	coverage time, sex, race, income, education, BMI, smoking, CESD-10 score and comorbidity count
Shen et al./2014/China [21]	General population N=20730 Age: 65.9; M(64.1%)	ICD-9	7.7	Incidence N=896	HR: 5.38 (4.52-6.40) no DM 4.05(3.26-5.03) DM	Age, sex, urbanization, income and co-morbidities of pneumoconiosis, interstitial lung disease, and pulmonary TB
Leung et al./2012/China [20]	N=62529 Aged >65 years; M (35.5%)	Physician diagnosed	8.3	Mortality N=1297	HR: 1.86 (1.58–2.19)	Age, sex, BMI, marital status, education, housing, alcohol, family history of malignancy
Fan et al./2011/China [19]	tin miners of YTC N=9295 Aged >40 years; M (93.6%)	Self-report	1992-2001	Incidence N=502	HR: 1.50 (1.24–1.81) CB	Age, sex, education, smoking status, pack-years, occupational radon and arsenic exposure and prior pulmonary disease
de Torres et al./2011/USA [18]	BODE, N=2507; Age: 65 M (92%)	DL_CO<80% FEV1<70%_	5	Incidence N=250	HR:1.76 (1.15–2.69)	Age, sex, BMI, pack-year history, smoking status, GOLD stages, DL_CO_, IC/TLC
Rodriguez et al./2010/UK [17]	GPRD: N=1927 Control N=16546 Age:40-89, M (72.3%)	Medical record	3.2	Incidence N=130	HR: 3.95 (2.39-6.52)	Age, sex, calendar year, smoking, PCP visits, referral and hospitalizations, ischaemic heart disease and depression.
Mortensen/2010/USA [15],	a previous diagnosis of pneumonia N=40 744 Aged ≥65 years M (98.1%)	ICD-9	1.5	Incidence N=3760	HR: 1.12 (1.04–1.20)	Age at admission, race/ethnic group, tobacco use and marital status
van Gestel et al/2009/ Netherlands [16]	N=3371 patients with peripheral arterial disease Age: 66; M (73%)	FEV1	5	Mortality N=102	HR: 2.06(1.32-3.20)	Age, gender, type of surgery, diabetes, smoking, hypercholestrolaemia, corticosteroids, statins and aspirin
Wilson et al./2008/USA [14]	N=3642, Age 50-79 years M (51.4%)	FEV1 Or on CT	3.7	Incidence N=99	OR: 2.09 (1.33–3.27) COPD 3.56(2.21–5.73) E	Age, sex, cigarette smoking, smoking dose intensity
Slatore et al./2008/ USA [46]	VITAL, N=77126 Age 50-76 years; M (48%)	Self-report	4.05	Incidence N=521	HR 1.45 (1.13–1.87)	Age, sex, years of smoking, pack-years, pack-years squared
Purdue et al./2007/Sweden [13]	Swedish construction workers N=176 997 M (100%)	FEV_1_	15	Incidence N=834	1.5 (1.2-1.9) mild 2.1 (1.7-2.6)moderate 2.7 (1.6- 4.6)severe	Age, smoking,
Turner/2007/USA [47]	CPS-II N= 448,600 M (27.1%)	Self-report	20	Mortality N=1759	HR: 0.96 (0.72-1.28) CB 1.66 (1.06-2.59) E 2.44 (1.22-4.90) COPD	Age, sex, race, education, marital status, BMI, occupational exposures, alcohol consumption, vegetable/fruit/fiber intake, fat intake, and passive smoking
de Torres et al./2007/Spain [12]	N=1166, Age 54 years M (74%)	FEV1 Or on CT	2002-2005	Incidence N=23	RR 2.89 1.14–7.27 COPD 3.13 (1.32–7.44) E	Age, sex, pack-years of smoking
Littman et al./2004/USA [11]	CARET: N=17 698 Age: 50-69 M (65%)	Self-report	9.1	Incidence N=1028	COPD HR 1.29 (1.09–1.53)	Age, sex, study arm, education, BMI, cigarettes smoked
Mannino et al./2003/USA [10]	NHANES I N=5402, Age:24-74 M (45.8%)	FEV1/FEV<70%	9.8	Incidence N=113	HR: 1.4(0.8-2.6) mild 2.8(1.8-4.4) moderate/ severe	Age, sex, race, smoking status, pulmonary function level, pack-years of cigarettes, years since last smoking
Vestbo et al./1991/Denmark [9]	N=876, Age 45-59 M (82.3%)	Self-report	1974-1985	Incidence N=35	0.8(0.27-2.45) CB	Age, tobacco consumption

Abbreviation: COPD, chronic obstructive pulmonary disease; MESA, Multi-Ethnic Study of Atherosclerosis; SCCS, Southern Community Cohort Study; LLPi, Liverpool Lung Project; CMS, Centers for Medicare and Medicaid Services; YTC, Yunnan Tin Corporation; BODE, Body Mass Index, Airflow Obstruction, Dyspnea, Exercise Performance; GPRD, General Practice Research Database, VITAL, VITamins And Lifestyle; CPS-II, Cancer Prevention Study II; CARET, Carotene and Retinol Efficacy Trial; NHANES I, National Health and Nutrition Examination Survey; HES, Hospital Episode Statistics; ICD, International Classification of Diseases; FEV, forced expiratory volume; CB, chronic bronchitis; CESD, Center for Epidemiologic Studies Depression Scale; D_LCO_, diffusion capacity for carbon monoxide.

### Meta-analysis

**Figure 2:**
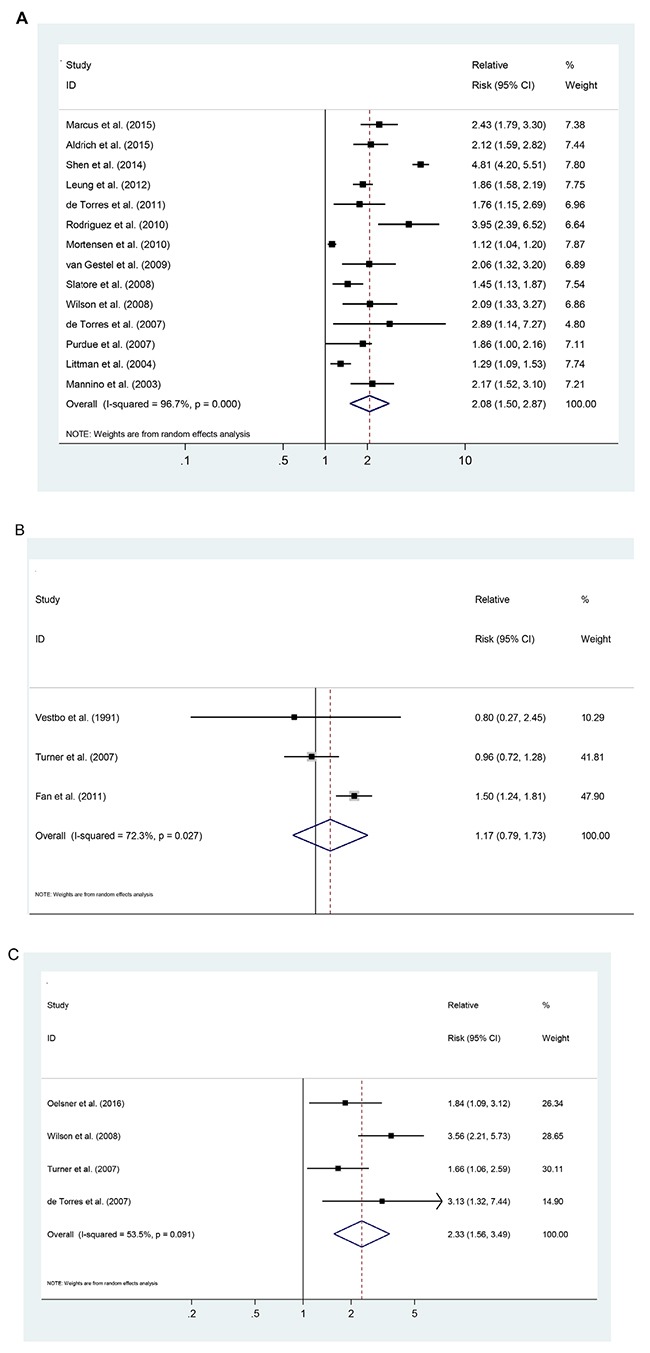
Pooled estimates of the risk associated with a previous diagnosis of chronic obstructive pulmonary disease (COPD), separated by condition **(A)** COPD. **(B)** chronic bronchitis. **(C)** emphysema.

Fourteen studies representing the association between COPD and lung cancer risk were used for this analysis. The SRR was 2.06 (95% CI: 1.50-2.85), and had high heterogeneity (P_heterogeneity_ <0.001, I^2^ = 96.7%; Figure 2A). There were three studies representing the risk associations for chronic bronchitis, with the SRR of 1.17 (95%CI: 0.79–1.73; P_heterogeneity_ =0.027, I^2^ = 72.3%; Figure 2B). Four studies represented the risk associations for emphysema, with the SRR of 2.33 (95% CI: 1.56–3.49; P_heterogeneity_ =0.091, I^2^ = 53.5%; Figure 2C).

### Subgroup analyses (shown in Table 2)

**Table 2:** Subgroup analyses of chronic obstructive pulmonary disease and lung cancer risk

	Studies, n	SRR (95% CI)	I^2^(%)	P_heterogeneity_	P_difference_
**All**	14	2.06 (1.50–2.85)	96.7	<0.001	
**Sex**					0.399
Male	6	2.04(1.11 –3.74	98.3	<0.001	
Female	3	2.67(1.27 –5.59)	93.3	<0.001	
**Locations**					**0.029**
Europe	4	2.40(1.81– 3.18)	49.3	0.116	
USA	8	1.65(1.32– 2.05)	84.0	<0.001	
Asia	2	2.99(1.18– 7.60)	98.7	<0.001	
**Smoking status**					0.289
Never	3	2.32 (1.42–3.77)	0	0.833	
Ever	3	1.60 (1.28-2.01)	76.0	<0.001	
**Diagnosis of COPD**					0.201
Physiological diagnosis	6	2.02(1.69 –2.41)	0	0.931	
Disease registry	3	2.75(0.87 –8.73)	99.4	<0.001	
Self-report	5	1.75(1.40 –2.19)	80.2	<0.001	
**COPD severity**					**0.016**
Mild	4	1.46 (1.20–1.76)	0	0.876	
Moderate	3	2.05(1.67–2.52)	8.1	0.337	
Severe	3	2.44(1.73–3.45)	0	0.438	
**Follow-up, years**					0.453
**≤5**	7	1.87 (1.36–2.59)	89.3	<0.001	
**>5**	7	2.23(1.41–3.53)	96.3	<0.001	
**Type of outcome**					0.948
Incidence	10	2.06 (1.34–3.16)	97.6	<0.001	
Mortality	4	1.95 (1.71–2.23)	0	0.775	

#### Study locations

When stratified by study locations, the strong association was observed in China (SRR =2.99, 95% CI: 1.18– 7.60, P _heterogeneity_<0.001), Europe (SRR =2.40, 95% CI: 1.81– 3.18, P _heterogeneity_ =0.116) and the USA (SRR =1.65, 95% CI: 1.32– 2.05, P _heterogeneity_ <0.001).

#### Gender

Most of the studies reported risk estimations for men and women combined, and three studies [20–22] reported specifically for men and women. Another three studies [13, 15, 18] consisted entirely or almost entirely of men (>90%). In stratified analyses by gender, COPD was associated with an increased risk of lung cancer in both men and women (men: SRR =2.04, 95% CI: 1.11 –3.74, P _heterogeneity_<0.001; women: SRR = 2.67, 95% CI: 1.27 –5.59, P _heterogeneity_ <0.001). There was no gender difference in the risk estimation (P =0. 289).

#### Smoking status

Three studies [11, 13, 20] reported risk estimations specifically for smoking status (never, ever, former and current smokers), and one study [47] involved only never smokers. The SRRs were 2.32 (95% CIs: 1.42–3.77) for never smokers, 1.60 (95% CIs: 1.28-2.01) for ever smokers, 1.63(95% CIs: 1.11-2.39) for current smokers and 1.32 (95% CIs: 0.65-2.68) for former smokers, respectively. The difference were not statistically significant among the smoking status (P =0.289).

#### Diagnostic method of COPD

We also performed subgroup analysis based on the methods of COPD diagnosis. The SRR in spirometry and radiographic evidence subgroup (SRR =2.02, 95% CI: 1.69 –2.41, P _heterogeneity_ = 0.931, I^2^=0) were similar with those in self-report (SRR =1.75, 95% CI: 1.40 –2.19, P _heterogeneity_<0.001, I^2^=80.2%) or disease registry subgroups (SRR =2.75, 95% CI: 0.87 –8.73, P _heterogeneity_<0.0071, I^2^= 99.4%).

#### COPD severity

Four studies [13, 14, 16, 45] presented results specifically for the severity of COPD, and the SRRs were 1.46 (95% CIs: 1.20–1.76; P_heterogeneity_ =0.876, I^2^= 0) for mild, 2.05(95% CIs: 1.67-2.52, P_heterogeneity_ =0.337, I^2^= 8.1%) for moderate and 2.44(95% CIs: 1.73-3.45, P_heterogeneity_ =0.438, I^2^= 0) for severe COPD, respectively.

#### Duration of follow-up

The SRRs were 1.87 (95% CIs: 1.36–2.59; P_heterogeneity_ <0.001, I^2^= 89.3%) for studies with a median duration of follow-up less than 5 years, which was similar with those for studies with a median duration of follow-up large than 5 years 2.23(95% CIs: 1.41-3.53, P_heterogeneity_ <0.001, I^2^= 96.3%).

#### Type of outcome

Among these 14 cohort studies, 10 used incidence rate ratios or hazard ratio as the measure of relative risk, while four used mortality rate ratios or hazard ratio [16, 20, 22, 47]. The SRR for lung cancer incidence with history of COPD was similar with that for lung cancer mortality (Incidence: SRR =2.06, 95% CI: 1.34 –3.16, P _heterogeneity_<0.001; mortality: SRR = 1.95, 95% CI: 1.71 –2.23, P _heterogeneity_ =0.775).

#### Histological subtype

Two cohort studies [11, 13] reported results on the association between COPD and histological subtype of lung cancer. This association was significantly positive for squamous cell cancer (SRR =1.85, 95% CI: 1.34–2.54, P_heterogeneity_ =0.147), but null for adenocarcinoma (SRR =1.25, 95% CI: 0.93– 1.68, P _heterogeneity_ =0.225) and small cell cancer (SRR =1.39, 95% CI: 0.74–2.61, P_heterogeneity_ =0.041).

### Sensitivity and meta-regression analysis

Exclusion of studies in which all subjects were reported with a previous diagnosis of pneumonia [15], peripheral arterial disease [16], or from specific occupation [11, 13] did not change the overall risk estimate for lung cancer (SRR =2.35, 95% CI: 1.69– 3.26). We also conducted a sensitivity analysis by omitting one study at a time and calculating the SRRs for the remainder of studies, and found that there were no changes in the direction of effect when any one study was excluded (Supplementary Figure 1).

We conducted meta-regression analyses to investigate the impact of above study characteristics on the risk estimates of lung cancer. Only study locations (P=0.029) was found to be the significant factor, and the heterogeneity explained by the population source was 36.3%.

### Publication bias

**Figure 3:**
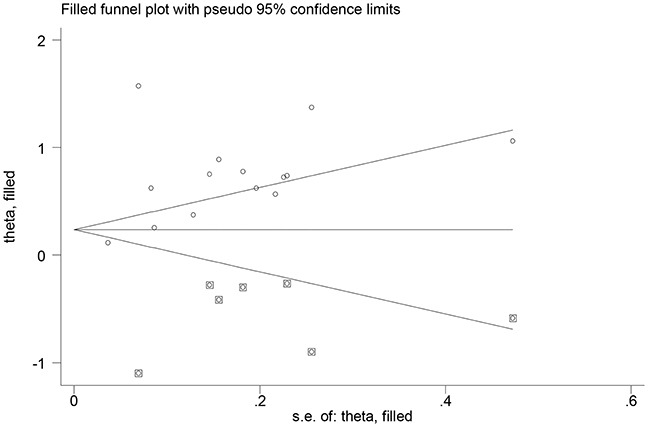
Filled funnel plot of log relative risk vs. standard error of log relative risks in studies that evaluated the effect of chronic obstructive pulmonary disease on the risk of lung cancer

Begg's (*P* =0.511) and Egger's (*P* =0.356) tests did not reveal evidence of publication bias, but visual inspection of the funnel plots revealed significant asymmetry. The trim-and-fill method suggested that 7 additional risk estimates were needed to balance the funnel plot, and the summary risk estimates were still statistically significant, albeit weaker (SRR =1.37; 95% CI, 1.01–1.92; Figure 3). We did not evaluate the publication bias for the association of emphysema and chronic bronchitis due to the small number of studies included.

## DISCUSSION

Based on eighteen prospective cohort studies, our meta-analysis provides the most comprehensive evidence that a significantly increased risk of lung cancer was observed for a history of COPD, emphysema, but not for chronic bronchitis. In addition, increased risk of lung cancer for COPD was seen for both men and women and among all the location subgroups. Importantly, the increased risk was significantly higher for squamous cell cancer than those for adenocarcinoma and for small cell cancer, and was COPD severity dependent.

Several mechanisms have been suggested to explain the predisposition of patients with COPD to develop lung cancer. First, mucociliary dysfunction caused by COPD may allow carcinogens from the smoke or other environmental substances in the mucous blanket to have longer exposure time at these sites, leading to development of lung cancer [48]. Secondly, airway obstruction in COPD may lead to chronic inflammation, which can result in increase the likelihood of the conversion of endogenous DNA damage into mutations. Thirdly, an imbalance between oxidants and antioxidants can lead to free radical damage of DNA [49]. Fourthly, there may be some genetic predisposition to both diseases as the familial clustering of pulmonary dysfunction in relatives of patients with lung cancer and in patients with COPD [50].

Both chronic bronchitis and emphysema can lead to chronic airway obstruction and then develop into COPD. Our finding showed that emphysema, but not chronic bronchitis, was a risk factor for the development of lung cancer. Results from a prospective cohort [12] of a lung cancer screening study showed that emphysema, but not airflow obstruction, is an independent risk factor for lung cancer. Wilson *et al*. [14] reported that although emphysema on CT scan and airflow obstruction on spirometry are related to lung cancer in a high-risk population, the former had a higher risk for lung cancer compared to the latter. By contrast, other studies [51, 52] found that emphysema was not a significant risk for lung cancer. Given the observational evidence and the low numbers of lung cancer events, these results may be prone to type II error.

In the current report, we found that the pooled risk association between COPD and lung cancer risk was severity dependent. That is, lung cancer incidence increased in a stepwise manner from the severity of COPD. A study from Calabro et al. [53] provided accurate quantification that lung function impairment was associated with a significantly increased risk of lung cancer: a reduction of as little as 10% of FEV_1_% pred is associated with an almost three-fold greater lung cancer risk. A recent meta-analysis [54] revealed that reduced FEV1 is strongly associated with risk of lung cancer, especially among women. Importantly, authors found that the risk relationship is dose- dependant. However, a study by de Torres et al [18] reported that incidence density of lung cancer is higher in COPD patients with milder airflow obstruction (19.9 per 1,000 person-yrs) than that with severe airflow obstruction (9.2 per 1,000 person-yrs). Authors speculated that patients with severe COPD have an active, non-tolerant, immune system, which would act as a barrier for the development and progression of lung cancer.

Analyses stratified by lung cancer histology can offer insight into the relationship between COPD and lung cancer. In the current meta-analysis, although not statistically significant, COPD cases had an increased risk of squamous cell carcinoma, small cell cancer and adenocarcinoma of the lung (SRR, 1.85 vs. 1.39 vs. 1.25). In contrast to the well established finding that smoking is a stronger risk factor for squamous cell carcinoma and small cell carcinoma than for adenocarcinoma of the lung [55], our findings for COPD showed a different pattern. Papi et al. [56] have reported that COPD might increase the risk of the squamous cell cancer by more than four times (OR=4.05, 95%CI, 1.93 to 10.57). These differences by histology support the notion that tobacco smoke may exert effects on the association between COPD and lung cancer. However, these results should be interpreted with caution because the current sub-analysis was based on only two studies. Further studies are warrant to clarify the association between COPD and lung cancer histology.

Our study has several strengths as follows: (1) we were able to update a number of recently published studies on the COPD-lung cancer association; (2) we could derive risk estimates with high levels of precision and the less possibility of recall or selection bias, because our analysis was according to eighteen prospective cohort studies, involving more than 10,000 lung cancer cases and 9 million participants; (3) We performed several sensitivity analysis based on sex, geographic locations, smoking status, exposure assessment, COPD severity, duration of follow-up, lung cancer histology in the COPD-lung cancer association.

There are several design and methodological limitations of this study that must be considered in the interpretation of the results. First, this meta-analysis detected great heterogeneity across studies. We performed meta-regression analyses showed that study locations have modified effects on this association, which might partially (36.3%) account for the high heterogeneity among studies.

Second, misclassification bias may exist. A history of COPD was ascertained by self-report in four studies, which lead to the attenuation of risk estimates and the true COPD-lung cancer association may be stronger than we observed. Although the precise definitions and diagnoses of COPD vary, some studies [57, 58] have attempted to verify self-reported history of COPD by the presence of airflow limitation due to chronic bronchitis and emphysema, with a sensitivity of 12.5%[58] to 78% [57]. Indeed, a sensitivity analysis by stratifying the total studies based on the methods of COPD diagnosis did not find a significant difference in the SRRs among methods of COPD assessment.

Third, because of the observational nature of our analysis, unmeasured and residual confounding is likely to be present. It has been debated that the COPD-lung cancer relationship may be a product of residual confounding by tobacco smoking, the shared risk factor for both COPD and lung cancer [44]. In our meta-analysis, we included only studies that controlled for the effects of smoking (status, intensity and duration). We further performed analyses according to smoking status, and found that the increased COPD-lung cancer association was independent of smoking status and the magnitude of the association was similar between never and ever smoking. Additionally, some confounding factors were not considered, such as biofuel smoke and second hand smoke (SHS), which is a shared risk factor for lung cancer [59] and COPD [60].

Forth, the effect of medications for COPD on this association should also be taken into account. To date, results from observational studies have suggested the potential that inhaled corticosteroids (ICS), which are commonly prescribed to COPD patients, may exert a protective role against the development and progression of lung cancer, particularly at high doses [61]. However, no studies involved in this meta-analysis considered the effects of ICS on the association between COPD and the risk of lung cancer.

Finally, the possibility of publication bias is of a concern, because small studies with null results tend not to be published. Although Begg's and Egger's tests did not reveal evidence of publication bias, visual inspection of the funnel plots did reveal significant asymmetry. The trim-and-fill method suggested 7 additional risk estimates were needed to balance the funnel plot, and the summary risk estimates were still statistically significant, albeit weaker (SRR =1.37).

In conclusion, the present study suggests a significantly increased risk of lung cancer associated with history of COPD. However, the possibility that the association may be due to bias or confounding cannot be completely excluded.

## MATERIALS AND METHODS

### Data sources and study identification

Two investigators (ZXY and JN) independently screened the original articles published in English language in MEDLINE and EMBASE databases from inception to December 31, 2016. We used the list of keywords in the form of following MeSH terms and Text Words: 1) COPD OR chronic obstructive pulmonary disease OR emphysema OR chronic bronchitis; 2) lung cancer OR lung neoplasm; 3) risk OR incidence OR prevalence OR mortality. References of selected retrieved articles were also manually searched. We did not consider abstracts or unpublished reports.

### Study selection

COPD is a term generally used to describe two end phenotypes: emphysema (the enlargement and destruction of the alveoli) and chronic bronchitis (chronic inflammation and scarring of bronchi), and the majority of patients with COPD are found somewhere in the middle. The most common definitions of COPD involve either airflow limitation (American Thoracic Society) or reduced maximum expiratory flow (European Respiratory Society). The present meta-analysis was based on estimates reported for these three conditions combined as well as reported separately (i.e., COPD, emphysema, chronic bronchitis).

Two authors (ZXY and JN) independently reviewed all the retrieved studies to determine if they meet the inclusion criteria and disagreements were settled through consensus with a third investigator (WLJ). The inclusion criteria were as follows:

(1) published as original prospective cohort studies reporting incidence or mortality of lung cancer after the diagnosis of COPD (including emphysema and/or chronic bronchitis);

(2) reported findings in terms of relative risk (RR), hazard ratio(HR), with corresponding 95% CIs or that reporting sufficient data to calculate them;

(3) adjusted or matched the risk estimations with age and smoking at least.

Non peer-reviewed articles, animal and mechanistic studies, ecologic assessments and correlation studies were not included for analysis. In case of several publications describing the same study, only the most recent or informative publication was included.

### Data extraction

The following information was extracted from each study by two researchers (ZXY and JN) independently: last name of the first author, study design, publication year, geographic locations, study size, study population, methods of COPD and lung cancer ascertainment, follow-up duration, the RR estimates with their 95% CI and adjustment variables. From each study, the risk estimates were extracted that have been adjusted for the greatest number of potential confounders. To ensure the accuracy of data extraction, this process was independently performed by three authors (JN, ZXY and WLJ). Any data discrepancy was also resolved by consensus.

### Statistical methods

Data analysis was performed using STATA, version 11.0 (STATA, College Station, TX, USA) statistical software. A two-tailed P value of <0.05 represents significance. In light of the high likelihood of between study variance, we used a random-effect model rather than a fixed-effect model. This method of a random-effects model was developed by DerSimonian and Laird [62]. Because mortality among lung cancer patients is quite high (5-year survival ~10-15% among western populations), we used mortality as a proxy measure for incidence. We used a fixed effects model to obtain overall combined estimates for lung cancer risk for studies that reported results separately for diabetes and non-diabetes [21], COPD severity [13, 45]. For studies reporting risk estimations for chronic bronchitis, emphysema and/or COPD [12, 14, 47], we used them in the specific meta-analysis.

Heterogeneity was assessed by Cochran *Q* and *I*^2^ statistics. P value of < 0.10 represents statistically significant heterogeneity. *I*^2^ values quantify the proportion of total variation across studies that is due to heterogeneity rather than chance, with the low, moderate, and high of 25%, 50%, and 75% respectively. Where the *I*^2^ value was 25% or lower, indicating no evidence of heterogeneity [63]. Sources of heterogeneity were explored using subgroup analyses and meta-regression analysis. We also investigated the influence of a single study on the overall risk estimate by sequentially removing study to test the robustness of the main results. Where possible, potential publication bias was assessed by visual inspection of the funnel plots and the further Begg's adjusted rank correlation and Egger's regression asymmetry test [64, 65]. The *P* values of <0.10 indicates potential publication bias. In addition, we evaluated the number of unpublished studies that would have to exist to negate the results and the pooled RR adjusted for publication bias using the trim and fill method [66].

## SUPPLEMENTARY MATERIALS FIGURES



## Abbreviations

COPD: chronic obstructive pulmonary disease
GBD: Global Burden of Disease
FEV: forced expiratory volume
CB: chronic bronchitis
RR: relative risks
CI: confidence interval
HR: hazard ratio
