# Supplementary material for: Chronic obstructive pulmonary disease and risk of lung cancer: a meta-analysis of prospective cohort studies
Source: Oncotarget. 2017 Aug 18;8(44):78044–56. doi: 10.18632/oncotarget.20351 (PMC5652835; doi:10.18632/oncotarget.20351)
Supplement: Supplementary file 1 [file oncotarget-08-78044-s001.pdf]

Chronic obstructive pulmonary disease and risk of lung cancer: a meta-analysis of prospective cohort studies

SUPPLEMENTARY MATERIALS

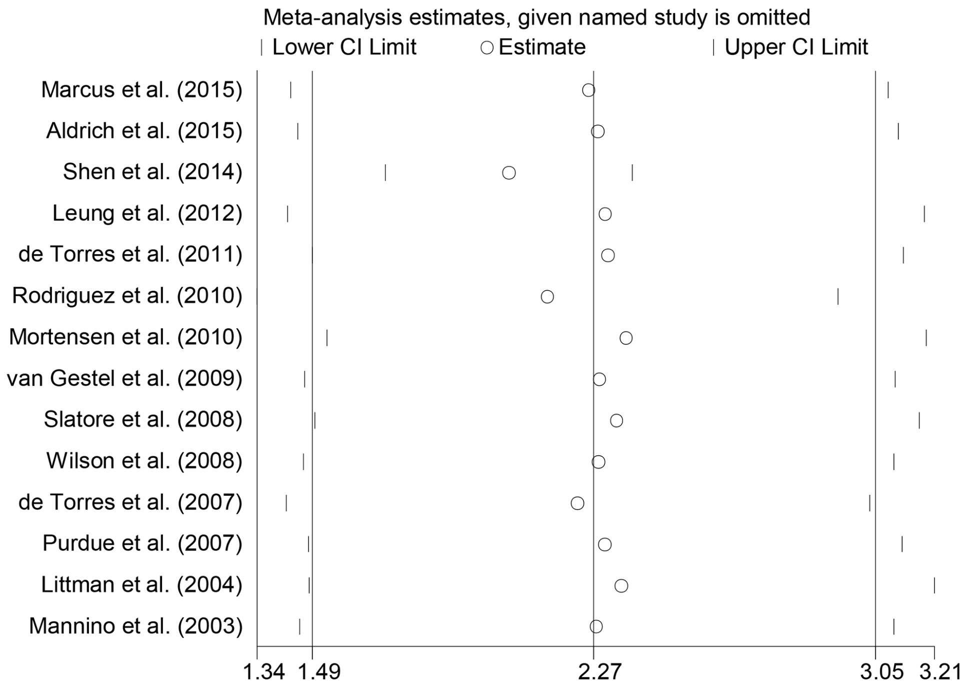

Supplementary Figure 1: Influence of removing studies one by one on the association between chronic obstructive pulmonary disease and lung cancer risk. Circles are effect estimates and horizontal dotted lines were 95% confidence intervals for meta-analysis of the remained studies; the vertical line in the center is the pooled effect estimate for all studies.
